# A Comparative Analysis of Feeding and Trophic Level Ecology in Stingrays (Rajiformes; Myliobatoidei) and Electric Rays (Rajiformes: Torpedinoidei)

**DOI:** 10.1371/journal.pone.0071348

**Published:** 2013-08-01

**Authors:** Ian P. Jacobsen, Mike B. Bennett

**Affiliations:** School of Biomedical Sciences, The University of Queensland, St. Lucia, Queensland, Australia; Aristotle University of Thessaloniki, Greece

## Abstract

Standardised diets and trophic level (*T*_L_) estimates were calculated for 75 ray species from the suborders Myliobatoidei (67 spp.) and Torpedinoidei (8 spp.). Decapod crustaceans (31.71±3.92%) and teleost fishes (16.45±3.43%) made the largest contribution to the standardised diet of the Myliobatoidei. Teleost fishes (37.40±16.09%) and polychaete worms (31.96±14.22%) were the most prominent prey categories in the standardised diet of the suborder Torpedinoidei. Cluster analysis identified nine major trophic guilds the largest of which were decapod crustaceans (24 species), teleost fishes (11 species) and molluscs (11 species). Trophic level estimates for rays ranged from 3.10 for *Potamotrygon falkneri* to 4.24 for *Gymnura australis, Torpedo marmorata* and *T. nobiliana*. Secondary consumers with a *T*_L_ <4.00 represented 84% of the species examined, with the remaining 12 species (16%) classified as tertiary consumers (*T*_L_ ≥4.00). Tertiary consumers included electric rays (*Torpedo,* 3 spp. and *Hypnos*, 1 sp.), butterfly rays (*Gymnura*, 4 spp.), stingrays (2 spp.) and Potamotrygonid stingrays (2 spp.). Feeding strategies were identified as the primary factor of influence with respect to Myliobatoidei and Torpedinoidei *T*_L_ estimates with inter-family comparisons providing the greatest insight into Myliobatoidei and Torpedinoidei relationships.

## Introduction

The increased level of information on elasmobranch (Chondrichthyes: Elasmobranchii) diets has seen a shift away from broad generalisations characterising all elasmobranchs (sharks, skates, rays) as apex predators to more quantitative multi-species dietary assessments [1]–[3]. Cortés presented [1] standardised diets of 149 shark species in order to determine the trophic level (*T*_L_) for each species and how these related to other top-order predators. A similar analysis was undertaken by Ebert & Bizzarro [2] for 60 species of skate (Rajiformes: Rajoidei). In so doing, both studies provided a more holistic account of how elasmobranchs influence regional ecosystems. In comparison, there is a lack of synthesis of the considerable dietary information available [3]–[8] for stingrays (Suborder: Myliobatoidei) and electric rays (Suborder: Torpedinoidei), and little information on their trophic relationships.

The Myliobatoidei is second largest suborder within the Rajiformes and comprises a morphologically diverse group of seven families, three subfamilies and over 200 recognised species [9]–[11]. Maximum body size (disc width, *W*_D_) varies considerably within and between families, from about 15 cm *W*_D_ in the Urolophidae (stingarees) and Urotrygonidae (round rays) to about 700 cm *W*_D_ in the Myliobatidae (subfamily Mobulinae – manta rays) [12]. While there are notable exceptions, such as the giant stingaree *Plesiobatis daviesi* (Wallace, 1967), species from the Myliobatoidei tend to inhabit relatively shallow, warm temperate to tropical waters and are particularly common within the Indo-West Pacific region [13]. In contrast, skates are more prominent in deeper, colder waters, particularly at higher latitudes [9].

The Torpedinoidei is the third largest suborder within the Rajiformes [9], and comprises four families and about 70 recognised species [10], [11]. The majority of species inhabit continental shelf waters to depths of 100 m in both tropical and temperate environments [13], [14]. Characterised by the presence of two well-developed electric organs, electric rays display some of the more unique prey capture techniques, stunning or killing prey with an electrical discharge [14], [15].

Dietary studies involving the Myliobatoidei and Torpedinoidei are often restricted to individual species with interspecific comparisons focusing principally on results obtained from shared analytical techniques *i.e.* comparisons of Index of Relative Importance (*I*_RI_) values. As a consequence, there is limited understanding of how the diets of ray species relate to each other and to the diets of other marine predators. The following study provides standardised dietary compositions and *T*_L_ estimates for a wide range of species from the suborders Myliobatoidei and Torpedinoidei. Designed to augment previous studies [1]–[3], the results obtained provide a significant contribution to the overall understanding of what trophic levels elasmobranchs occupy and how these relate to other marine predators. The study also provides a comprehensive overview of the available dietary data for each of the suborders and represents the first detailed *T*_L_ analysis involving multiple electric ray species.

## Materials and Methods

Order and suborder classifications for the study were based on Ebert & Compagno [9] with family classifications based of Eschmeyer & Fong [10]. For the purpose of the analysis, species from the family Myliobatoidei were grouped into their subfamilies (Myliobatinae, Rhinopterinae and Mobulinae) and treated as distinct entities. Diet standardisation and *T*_L_ calculations were performed in accordance with Cortés [1] and Ebert & Bizzarro [2] with quantitative dietary data summarized from peer-reviewed journal articles, graduate theses and grey literature. The *Web of Knowledge* search engine was used to identify studies of relevance; followed by an examination of citations within the literature to identify additional sources of information. All studies included in the analysis are available either through the relevant literature database or in the case of graduate theses and grey literature, the institution where the research was undertaken. A full list of the references used to calculate the standardised diets and *T*_L_ estimates is provided in the supporting information (Appendix S1).

In order to calculate the standardised diets and estimate the *T*_L_ for each species, prey items within each of the respective sources were initially reviewed and grouped into 11 general categories (Table 1). All but one of the 11 prey categories were assigned the same prey trophic level (*T*_LP_) estimate as used by Ebert & Bizzarro [2]. As *T*_LP_ values for Protochordates (PROT: Cephalochordata and Tunicata) were not available the *T*_LP_ value for molluscs (MOLL) was used as a proxy [3]. In addition, squid and other cephalopod prey items were represented under the one category as they have the same *T*_LP_ value (Table 1) [2].

**Table 1:** Prey categories used to calculate standardised diet compositions and trophic levels – compiled from Cortés [1] and Ebert & Bizzarro [2].

Prey Category	Inclusions/Exclusions within each Prey Category	Trophic level (*T*_L_)
MOLL	Molluscs (excluding Cephalopoda), includes unidentified molluscs	2.1
PROT	Protochordates, includes *Amphioxus* and acorn worms	2.1
EUPH	Euphausiidae, Mysida, and other zooplankton	2.25
CRUS	Crustaceans (other than elsewhere specified), includes Stomatopoda, andunidentified crustaceans	2.4
INV	Invertebrates (other than elsewhere specified), includes unidentifiedinvertebrates and insects	2.5
DECA	Brachyura, Caridae, Penaeidae, Palinura	2.52
POLY	Polychaetes and other marine worms	2.6
AMPH	Amphipoda, Isopoda	3.18
CEPH	Cuttlefish, squid, octopus, and unidentified cephalopods	3.2
FISH	Fishes (other than chondrichthyans)	3.24
ELAS	Sharks, skates and rays	3.65

When more than one dietary study was available for a species or when dietary data were only reported for size classes, an index of standardised diet composition (*P_j_*) was calculated for each prey category. This index is weighted to account for differences in sample size and is calculated using the equation:
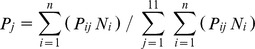
where *P_ij_* is the proportion of prey category *j* in study *i*, *N_i_* is the number of stomachs with food used to calculate *P_ij_* in study *i*, *n* is the number of studies, *j* is the number of prey categories (11) and Σ*P_j_* = 1 [1]. Where possible, *P_ij_* was based on compound indices such as the *I*_RI_ [16], [17], index of absolute importance [18], or index of preponderance [19]. When a compound index was not available, but more than one index was presented, a geometric index of importance was calculated by averaging the values *e.g.* (%N+%W)/2 [20]. Single indices including percent frequency of occurrence (%F_o_, %O), percent number contribution (%N_c_), percent volumetric contribution (%V_c_) or percent weight contribution (%W_c_) were only used when multiple indices were not available [2].

The *T*_L_ of each of species was calculated using:
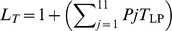
where *T*_LP_ is the trophic level of the prey category, *j* and *P_j_* are the contributions each prey category made to the diet of each species [1], [2].

Frequency of prey occurrence (*i.e.* presence/absence), standardised diets and individual *T*_L_ estimates were calculated for all 75 stingray and electric ray species. An average *T*_L_ and standardised diet was also calculated for each of the respective families and suborders. Calculation of a precision estimate to determine sample size sufficiency for the inclusion of a species in family and suborder level calculations was generally compromised by insufficient information in the source literature [2], [21]. Further, restricting the scope of the analyses to studies where sample size had been demonstrated to be sufficient through precision estimates (*i.e.* through cumulative prey curves) [21] would have resulted in a significant amount of data being omitted from the analysis. Given this, the approach taken by Cortés [1] and Ebert & Bizzarro [2] was adopted with a minimum sample limit of 20 stomachs set for the inclusion of a species in family and order level calculations. The 20 stomach limit has been used successfully in previous elasmobranch trophic level analyses [1], [2] and is designed to enhance the robustness of conclusions drawn and minimise the influence of species with smaller sample sizes [1]. Of the 75 species, 66 had 20 or more stomachs sampled and were subsequently included in the family and suborder average diet and trophic level calculations.

When applicable, a one-way analysis of variance (ANOVA) was used to test whether *T*_L_ varied significantly between suborders and families/subfamilies. The Tukey Test [22] was applied post-hoc for all pairwise comparisons of normalised data. Where data were non-normal and could not be normalized, a Kruskal-Wallis one-way ANOVA on ranks was used to compare *T*_L_ variability with Dunn’s test [23] applied post-hoc for all pairwise comparisons. In addition, a cluster analysis was undertaken using the PRIMER v5.0 package [24] and incorporated all species with samples greater than 20 stomachs. In this instance, the Euclidean Distance (*D*_E_) was assigned as the measure of dissimilarity with dissimilarity measures greater than 50% of the maximum overall *D*_E_ considered to be indicative of a major division. These values were used to distinguish between trophic guilds [2]. All descriptive statistics were compiled using SigmaStat (v.2.03 S.P.S.S. Inc.) with significance accepted at *P*<0.05. All means are presented with the standard error and only include species with stomach samples greater than 20 [1], [2].

## Results

Standardised diets and *T*_L_ estimates were calculated for 67 Myliobatoidei species and eight Torpedinoidei species. The Myliobatoidei subsample included eight families and 17 genera, compared with four families and four genera for the Torpedinoidei. At the family/subfamily level, the Dasyatidae (stingrays) were represented by the greatest number of species with 26 (Table 2; Table S1). Two families, the Narkidae and the Hypnidae were each represented by a single species. Quantitative dietary data were not available for the Myliobatoidei families Plesiobatidae (deepwater stingray) and Hexatrygonidae (sixgill stingray).

**Table 2:** Average prey contributions in the standardised diets for the suborders Myliobatoidei and Torpedinoidei and taxonomic families based on species with samples greater than 20 stomachs.

	SP	N	n	DECA	AMPH	EUPH	CRUS	MOLL	CEPH	INV	FISH	PROT	POLY	ELAS
**Overall**	**66**	**117**	**15380**	**28.12**	**6.55**	**3.90**	**6.91**	**13.38**	**2.08**	**4.05**	**18.96**	**0.27**	**15.66**	**0.10**
**Myliobatoidei**	**59**	**104**	**14071**	**31.71**	**6.62**	**4.30**	**7.50**	**14.49**	**1.29**	**3.83**	**16.45**	**0.30**	**13.47**	**0.05**
Dasyatidae	25	44	6515	46.16	2.38	0.86	6.18	10.77	2.00	1.36	15.50	0.61	14.09	0.09
Gymnuridae	4	6	942	3.22	0.00	0.00	3.19	2.56	1.45	0.06	89.50	0.00	0.03	0.00
Potamotrygonidae	4	8	470	18.43	11.49	0.00	24.72	0.76	0.00	17.80	26.79	0.00	0.01	0.00
Urolophidae	9	12	1166	20.52	28.72	6.87	7.42	0.32	0.16	1.23	1.19	0.21	33.35	0.00
Urotrygonidae	6	11	1812	52.82	5.13	10.82	9.19	2.15	0.00	1.62	1.69	0.05	16.53	0.00
Myliobatinae	8	16	2697	10.10	1.18	0.00	6.26	60.22	2.40	2.26	11.55	0.00	5.95	0.08
Mobulinae	1	1	52	0.00	0.01	99.68	0.31	0.00	0.00	0.00	0.00	0.00	0.00	0.00
Rhinopterinae	2	6	276	7.76	5.00	0.86	0.99	36.43	0.00	40.47	2.23	0.30	5.96	0.00
**Torpedinoidei**	**7**	**14**	**1453**	**6.98**	**5.01**	**0.00**	**1.01**	**2.18**	**8.45**	**6.52**	**37.40**	**0.00**	**31.96**	**0.49**
Narcinidae	3	6	630	5.56	11.58	0.00	2.31	5.08	0.00	0.13	4.76	0.00	70.58	0.00
Torpedinidae	2	6	704	1.30	0.05	0.00	0.07	0.00	0.33	0.00	96.44	0.00	0.09	1.71
Narkidae	1	1	91	29.14	0.00	0.00	0.00	0.00	0.00	45.27	13.82	0.00	11.77	0.00
Hypnidae	1	1	25	0.46	0.21	0.00	0.00	0.00	58.51	0.00	40.82	0.00	0.00	0.00

SP = the number of species with samples greater than 20 stomachs; N, number of dietary data sets; n, total number of stomachs. Refer to Table 1 for prey category definitions.

The standardised diets of most species (56.0%) were characterised by use of a single dietary data set, with a further 26.7% based on two data sets (Table 2). The standardised diet of the common stingray *Dasyatis pastinaca* (Linnaeus, 1758) was based on the highest number of dietary studies (N = 5) and the largest stomach sample size (*n* = 1265, Table S1). The common eagle ray *Myliobatis aquila* (Linnaeus, 1758) was the only other species whose standardised diet was based on analysis of over 1000 stomachs. Six species (8.0%) had 500–1000 stomach samples; 35 species (46.7%) between 100 and 500 stomachs, 32 species (42. 7%) had fewer than 100 stomachs and the standardised diet of nine species were based on less than 20 stomachs. A full species list including standardised prey contributions and individual *T*_L_ estimates is provided in Table S1.

All eleven prey categories were recorded in the diet of at least one species. On a presence-absence basis decapod crustaceans (DECA) had the highest frequency of occurrence, being recorded in the diet of 88.0% of surveyed species. Teleost fishes (FISH: 81.3%) had the second highest frequently of occurrence followed by polychaetes (POLY: 74. 7%) and ‘other crustaceans’ (CRUS: 74.6%). At the subordinal level, DECA (88.1%), FISH (80.6%), CRUS (74.6%) and POLY (73.1%) were the most frequently observed prey categories in the diet of Myliobatoidei species. Within the Torpedinoidei the three most prominent prey categories were DECA, FISH and POLY with each recorded in the diets of 87.5% of the species examined.

**Figure 1:**
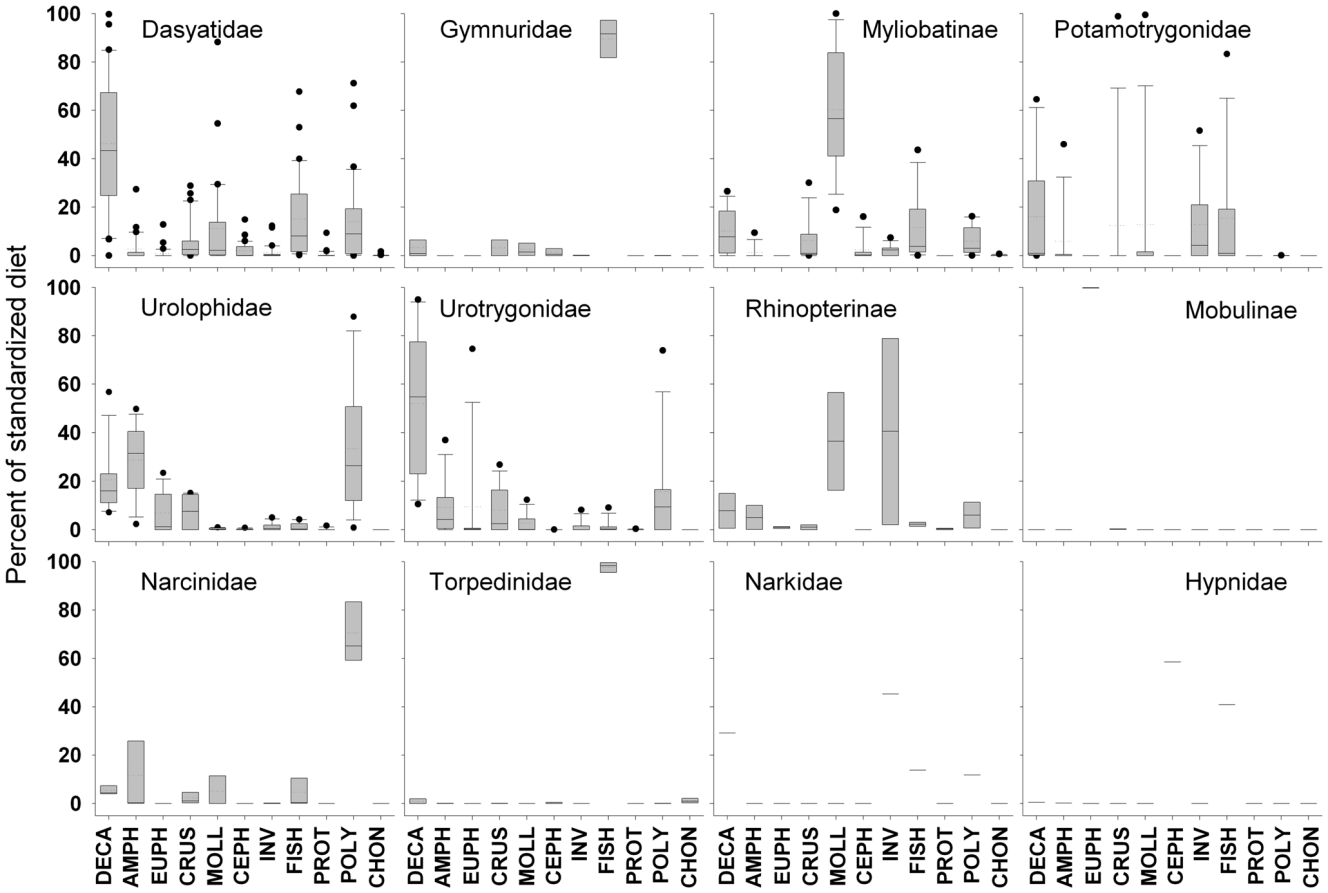
Prey category contributions to the standardised diets of each of the respective families and sub-families. Box plot represents the median standardised diet percentage (central line) and 25^th^ and 75^th^ percentiles; bars represent 10^th^ and 90^th^ percentiles; closed circles 5^th^ and 95^th^ Percentiles.

Sixty-six of the 75 species had over 20 stomach samples and were therefore included in the cluster analysis, average standardised diet calculations and average *T*_L_ estimates (Table 2). At the family level, the average standardised diet of the Dasyatidae was based on the highest number of quantitative dietary data sets (N = 44); approximately three times that recorded for the subfamily Myliobatinae (N = 16) and the Urolophidae (N = 12). Decapod crustaceans (DECA) was the main prey category in the averaged standardised diet of the Myliobatoidei (31.7±3.9%) followed by FISH and MOLL (Table 2). In comparison, approximately 70% of the standardised diet of the suborder Torpedinoidei consisted of FISH (37.4±16.1%) and POLY (32.0±14.2%). The dominance of the prey categories diversified at the family and subfamily level with DECA the most important prey category for the Dasyatidae and Urotrygonidae, MOLL for the subfamilies Myliobatinae and Rhinopterinae, FISH for Gymnuridae and Torpedinidae and POLY for Urolophidae, and Narcinidae. Cephalopod molluscs (CEPH), Euphausiids and mysids (EUPH) and other invertebrates (INV) were each identified as the most important prey category in the Hypnidae, Mobulinae and Narkidae respectively (Table 1–2; Fig. 1).

**Figure 2:**
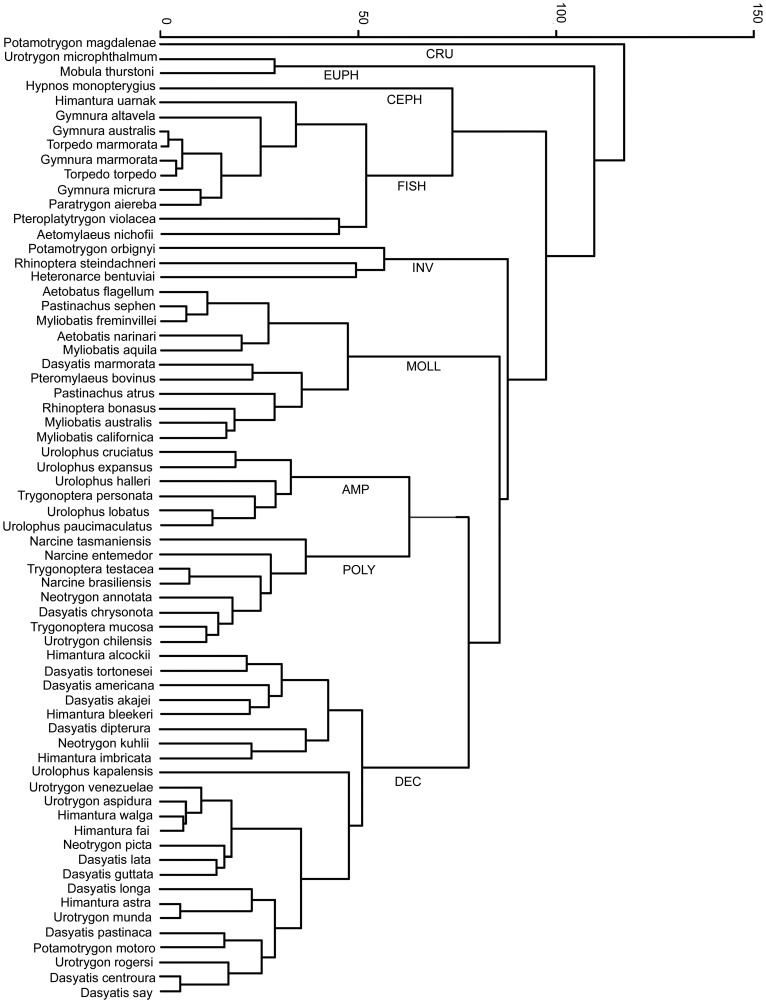
Cluster analysis of standardised diet compositions for Myliobatoidei and Torpedinoidei with >20 stomachs (*n* = 66).

Cluster analysis of standardised diets for species with >20 sampled stomachs (*n* = 66) revealed nine major trophic guilds and a maximum overall dissimilarity distance of 117.2 (Fig. 2). The diets of species within these guilds were dominated by the following prey categories CRUS, DECA, FISH, MOLL, POLY, CEPH, EUPH, INV, and amphipods and isopods (AMPH). The DECA trophic guild (*D*_E_ of 77.9) had the highest representation of the study with 24 species, followed by FISH (*D*_E_ = 73.7) and MOLL with 11 species (Fig. 2). The CRUS (1 species), CEPH (1 species) and EUPH (2 species) trophic guilds had the smallest representations of the study (Fig. 2).

At 3.90 (±0.12) the mean *T*_L_ for the Torpedinoidei was slightly higher than that of the Myliobatoidei (Table 3). At the family level the Torpedinidae (*T*_L_ = 4.24) and Hypnidae (*T*_L_ = 4.21) had the highest *T*_L_ values of this study; the subfamily Mobulinae had the lowest average *T*_L_ value at 3.25 (Table 3). The majority of species in the Myliobatoidei and Torpedinoidei (84%, 63 spp.) were identified as secondary consumers with a *T*_L_ of <4.0; the majority of which had a *T*_L_ value of between 3.50 and 3.99 (Table S1). The remaining 12 species (16%) were identified as tertiary consumers (*T*_L_ values ≥4.0) and included species from the families Gymnuridae (*n* = 4), Torpedinidae (*n* = 3), Dasyatidae (*n* = 2), Potamotrygonidae (*n* = 2), and Hypnidae (*n* = 1). The longheaded eagle ray *Aetobatus flagellum* (Bloch & Schneider, 1801) and the largespot river stingray *Potamotrygon falkneri* Castex & Maciel, 1963 had the lowest individual trophic level value of the study at *T*_L_ = 3.10. The Australian butterfly ray *G. australis* and two species of *Torpedo* had the highest individual *T*_L_ value of 4.24 (Table S1).

**Table 3:** Trophic levels of stingrays, electric rays, skates and sharks (updated from Ebert & Bizzarro) [2].

Taxon	Order/Family	SP	Mean	LCL	UCL	Min	Max
**Suborder**	**Rajoidei**	**60**	**3.8**	**3.7**	**3.8**	**3.5**	**4.2**
Family	Anacanthobatidae	1	3.5	**–**	**–**	3.5	3.5
Family	Arhynchobatidae	19	3.9	3.8	3.9	3.5	4.1
Family	Rajidae	40	3.8	3.7	3.9	3.5	4.2
**Suborder**	**Myliobatoidei**	**59**	**3.62**	**3.65**	**3.59**	**3.10**	**4.24**
Family	Dasyatidae	25	3.62	3.65	3.58	3.16	4.08
Family	Gymnuridae	4	4.16	4.20	4.12	4.05	4.24
Family	Potamotrygonidae	4	3.71	3.83	3.58	3.40	4.12
Family	Urolophidae	9	3.70	0.03	3.74	3.67	3.58
Family	Urotrygonidae	6	3.52	0.04	3.56	3.48	3.34
Subfamily	Myliobatinae	8	3.37	3.45	3.29	3.10	3.72
Subfamily	Mobulinae	1	3.25	**–**	**–**	3.25	3.25
Subfamily	Rhinopterinae	2	3.43	3.51	3.36	3.36	3.51
**Suborder**	**Torpedinoidei**	**7**	**3.90**	**4.02**	**3.78**	**3.59**	**4.24**
Family	Narcinidae	3	3.66	3.73	3.60	3.59	3.79
Family	Torpedinidae	2	4.24	4.24	4.23	4.23	4.24
Family	Narkidae	1	3.62	**–**	**–**	3.62	3.62
Family	Hypnidae	1	4.21	**–**	**–**	4.21	4.21
**Order**	**Carcharhiniformes**	**90**	**4.0**	**3.9**	**4.1**	**3.2**	**4.3**
Family	Carcharhinidae	39	4.1	4.1	4.2	3.8	4.3
Family	Hemigaleidae	2	4.2	4.1	4.3	4.3	4.3
Family	Proscyllidae	2	4.1	4	4.1	4	4.1
Family	Pseudotriakidae	1	4.3	**–**	**–**	4.3	4.3
Family	Scyliorhinidae	21	3.9	3.8	4	3.5	4.2
Family	Sphyrnidae	6	3.9	3.6	4.2	3.2	4.3
Family	Triakidae	19	3.8	3.7	3.9	3.5	4.2
**Order**	**Lamniformes**	**8**	**4**	**3.7**	**4.4**	**3.2**	**4.5**
Family	Alopiidae	2	4.2	4.2	4.2	4.2	4.2
Family	Cetorhinidae	1	3.2	**–**	**–**	3.2	3.2
Family	Lamnidae	3	4.3	4.2	4.5	4.2	4.5
Family	Megachasmidae	1	3.4	**–**	**–**	3.4	3.4
Family	Odontaspididae	1	4.4	**–**	**–**	4.4	4.4
**Order**	**Orectolobiformes**	**6**	**3.6**	**3.4**	**3.9**	**3.1**	**4.1**
Family	Ginglymostomidae	2	4	3.8	4.2	3.8	4.1
Family	Hemiscyllidae	2	3.6	3.5	3.8	3.5	3.7
Family	Rhincodontidae	1	3.6	**–**	**–**	3.6	3.6
Family	Stegostomatidae	1	3.1	**–**	**–**	3.1	3.1
**Order**	**Hexanchiformes**	**5**	**4.3**	**4.5**	**4.5**	**4.2**	**4.7**
Family	Chlamydoselachidae	1	4.2	**–**	**–**	4.2	4.2
Family	Hexanchidae	4	4.1	4.5	4.5	4.2	4.7
**Order**	**Pristiophoriformes**	**1**	**4.2**	**–**	**–**	**4.2**	**4.2**
Family	Pristiophoridae	1	4.2	**–**	**–**	4.2	4.2
**Order**	**Squatiniformes**	**6**	**4.1**	**4**	**4.2**	**4.0**	**4.2**
Family	Squatinidae	6	4.1	4	4.2	4.0	4.2
**Order**	**Squaliformes**	**32**	**4.1**	**4**	**4.2**	**3.5**	**4.4**
Family	Echinorhinidae	1	4.4	**–**	**–**	4.4	4.4
Family	Squalidae	31	4.1	4	4.2	3.5	4.3
**Order**	**Heterodontiformes**	**1**	**3.2**	**–**	**–**	**3.2**	**3.2**
Family	Heterodontidae	1	3.2	**–**	**–**	3.2	3.2

SP number of species; LCL, 95% lower confidence limit; UCL, 95% upper confidence limit.

When compared, no significant relationship was observed between *T*_L_ estimates and the dominate descriptors of body size. A weak, negative correlation was detected between *T*_L_ and maximum disc width for the Myliobatoidei species (Spearman rank correlation coefficient, r_s_ = −0.1167, *P*>0.05, n = 64). Similarly a weak but positive correlation was detected between *T*_L_ and Torpedinoidei total length (Spearman rank correlation coefficient, r_s_ = 0.1071, *P*>0.05, n = 8). Removal of filter-feeding species from the Myliobatoidei sample resulted in a marginal increase in the Spearman rank correlation coefficient (r_s_ = 0.1509, *P*>0.05, n = 61). The thorny round stingray *Urotrygon chilensis* (Günther, 1872), dwarf round stingray *U. nana* Miyake & McEachran, 1998 and munda round ray *U. munda* Gill, 1863 were not included in the Myliobatoidei analysis due to the unavailability of an accurate measurement of maximum disc width.

Statistical comparisons of *T*_L_ estimates between Myliobatoidei and Torpedinoidei species with more than 20 stomachs, detected a significant difference between the average *T*_L_ of the two suborders (ANOVA, *F* = 7.70, d.f. = 1, *P*<0.05). A significant difference was also detected between the average *T*_L_ of Myliobatoidei families (including subfamilies) (Kruskal-Wallis one-way ANOVA on ranks: *H* = 30.61, d.f. = 7, *P*<0.001). Pairwise comparisons (Dunn’s Method) between the average *T*_L_ estimates of Myliobatoidei revealed a significant difference (*P*<0.05) between the Mobulinae and both the Gymnuridae and Urolophidae (Table 3). A secondary comparison of Myliobatoidei *T*_L_ estimates with the Mobulinae and Rhinopterinae removed from the analysis; the two families with the smallest representation, also showed a significant difference (ANOVA, *F* = 11.70, d.f. = 5, *P*<0.05). Pairwise comparisons (Tukey Test) between the six remaining families showed that the average Gymnuridae *T*_L_ differed significantly from all other families: Gymnuridae *vs.* Myliobatinae (*q* = 10.2, *P*<0.001); Urotrygonidae (*q* = 7.99, *P*<0.001); Dasyatidae (*q* = 8.03, *P*<0.001); Urolophidae (*q* = 8.03, *P*<0.01); Potamotrygonidae (*q* = 4.60, *P*<0.05). The average Myliobatinae *T*_L_ also differed significantly from the Urolophidae (*q* = 5.48, *P*<0.01), the Potamotrygonidae (*q* = 4.943, *P*<0.05) and the Dasyatidae (*q* = 4.79, *P*<0.05). No statistical comparisons were made between the Torpedinoidei as three of the four families were represented by only one or two species (Table 3).

## Discussion

Quantitative dietary data were available for about 30% of species within the Myliobatoidei and 12% of species within the Torpedinoidei. A similar situation was observed in the Rajoidei (skates) where about 24% of the described species had quantitative dietary data [2]. Despite the relatively low proportion of species for which suitable dietary data were available, the majority of families in the Myliobatoidei and Torpedinoidei were represented by at least one species; the exceptions being the monotypic Plesiobatidae (represented by *P. daviesi*) and Hexatrygonidae (represented by the sixgill stingray *Hexatrygon bickelli* Heemstra & Smith 1980).

In comparison to the previous studies of shark and skate diets [1], [2], rays of the Myliobatoidei and Torpedinoidei averaged 1.69±0.12 dietary studies per species compared to 2.98±0.24 for sharks [1] and 2.07±0.23 for skates [2]. The difference in study effort is also markedly different, with the majority of ray species’ diets characterised through a single study and a maximum of five dietary studies for a single species (*D. pastinaca*). This is in contrast to nine studies for both the thornback skate *Raja clavata* Linnaeus, 1758 and thorny skate *Amblyraja radiata* (Donovan, 1808) and 17 for the spiny dogfish *Squalus acanthias* Linnaeus, 1758 [1], [2]. Similarly, the maximum number of stomachs sampled for a single species was 1,265 for *D. pastinaca* (current study); compared with 19,259 for *S. acanthias* [1] and 19,738 for the little skate *Leucoraja erincea* (Mitchill, 1825) [2]. It is noted though that all three studies contained a relatively high proportion of species with samples of fewer than 100 stomachs; 42.7%, present study; 51.0% sharks [1]; 38.3%, skates [2].

A likely factor contributing to the Myliobatoidei and Torpedinoidei having relatively few dietary studies and low sample sizes is the availability of specimens and the type and location of the fisheries they interact with. The three skate species with the highest sample numbers *L. erinacea* (19,738), *A. radiata* (8,381) and *R. clavata* (3,424) are all caught in the Atlantic Ocean and retained for human consumption or for use as lobster bait [25]–[27]. Likewise, *S. ancathias* from the Atlantic Ocean, Mediterranean Sea and Pacific Ocean is retained for commercial sale [28]. Importantly, these species are, at least in part, caught in well-developed and regulated fisheries such as those of the United States of America and the United Kingdom [25], [26]. This provides greater access and opportunity with respect to collection and processing of large sample sizes.

In contrast, the principal commercial markets for stingray species in the Indo-Pacific region tend to be artisinal fisheries [29], [30]. The most notable of these occur in the Indonesian Archipelago which is home to the largest chondrichthyan fishery in the world [31]. Dietary studies in these areas are often impeded by sampling costs, an inability to obtain fresh samples or an inability to adequately process samples *e.g.* freeze specimens for subsequent analysis. Furthermore, the Indonesian Archipelago has significant problems with respect to illegal, unreported and unregulated shark and ray fishing activity [29]. As a consequence, dietary studies have a low priority when compared to the quantification of catch rates, determination of population trends [29], [31] and enhancement of baseline biological information, such as growth rates and reproductive parameters [32]. While stingrays and electric rays are caught in commercial fisheries in Australia, their retention is often limited by legislation or low market demand [12], and thus this also affects the availability of specimens. Further, the collection of specimens for species that do not form part of a commercial catch is generally time consuming and costly.

While large sample sizes and multiple dietary studies are not necessary for the determination of *T*_L_ values, it does provide for more robust estimates and minimises the influence of additional dietary samples [1]. In the current study nine species had *T*_L_ values based on fewer than 20 stomachs; five of which had ≤10 stomachs analysed (Table S1). In these instances, the inclusion of additional dietary samples would probably result in a different and more accurate *T*_L_ value [1] for these species. For most of these species though, it is unlikely that changes in the *L*_T_ value would alter their categorisation from a secondary consumer to a tertiary consumer. However, more substantial changes might be expected for species such as for *P. falkneri* which had a relatively low sample number and a *T*_L_ value substantially lower than that reported for other members of the genus (Table S1).

When compared, *T*_L_ estimates for the Myliobatoidei and Torpedinoidei were within the range previously reported for elasmobranch orders (Table 3). At 3.62 (±0.03), the mean Myliobatoidei *T*_L_ was the lowest recorded for a batoid suborder/order and the third lowest when sharks and skates were also taken into account. In comparison, the Torpedinoidei had the highest average batoid *T*_L_ estimate (3.90±0.12) which was similar to the average *T*_L_ estimate for Carcharhiniformes and Lamniformes, and higher than that for Heterodontiformes and Orectolobiformes (Table 3). Both suborders however contained secondary and tertiary consumers with Myliobatoidei having a broader range (3.10 to 4.24 *T*_L_) when compared to the Torpedinoidei (3.59–4.24 *T*_L_). Similar levels of variance were observed in trophic level analyses involving shark [1] and skate [2] species (Table 3).

The often complex nature of elasmobranch feeding strategies [33] makes it difficult to make broad generalisations about the diets of stingrays and electric rays. For instance, the range of trophic level values for species within the Gymnuridae (*T*_L_ = 4.05–4.24) was well above the average for the Myliobatoidei as a whole (*T*_L_ = 3.62±0.03; Table 3). The primary reason for such variation is that species within the Myliobatoidei employ a range of feeding strategies from filter feeding mobulid rays to ambush predators [12]. Similar *T*_L_ value trends occur in some non-batoid elasmobranch groups, such as the Orectolobiformes which includes both demersal foraging and filter feeding zooplanktivorous species [12], [34] and has an average *T*_L_ value comparable to that of the Myliobatoidei (Table 3). Given the wide variation in feeding strategies at the order/suborder level, inter-family comparisons of both *T*_L_ values and average standardised diet compositions provide a better indicator of how elasmobranch trophic relationships differ.

Batoid feeding behaviours can effectively be divided into three broad strategies: a) continuous feeders or foragers, b) ambush predators and c) filter feeders. Continuous feeders tend to ingest small prey at fairly regular intervals resulting in high numbers of prey items in the stomach, prey items in varying stages of digestion and a low occurrence of empty stomachs [35]. Of the species where the standardised diets was based on >20 stomachs, 58 were considered to be continuous feeders including members of the Dasyatidae, Myliobatinae, Potamotrygonidae, Rhinopterinae, Urolophidae, Urotrygonidae, Narcinidae and Narkidae (Table S1, Fig. 1). These 58 species had an average *T*_L_ value of 3.59 (±0.03) and a standardised diet composition consisting predominantly of benthic prey items (DECA = 32.8±3.9%; POLY = 17.6±3.2%) which was reflected in the cluster analysis where the majority of species grouped together in the POLY and DECA trophic guilds (Fig. 2).

Continuous feeding, as defined above, is the strategy most frequently employed by stingrays and skates [3], [35]. These species typically ingest prey living on the surface of the substrate or utilise inertial suction to target prey buried in the immediate subsurface layer [33], [36], with larger species able to ingest larger, more mobile prey [37]. It is interesting that the diet of species within the Narcinidae and Narkidae appears to be more consistent with continuous feeding species, whereas species within the Torpedinidae and Hypnidae have a prey contribution profile consistent with that of the ambush predators (Table 2, Fig. 1). As all of these species possess two well-developed electrical organs [12], [38] it might be expected that the feeding strategies and diets of species within the Torpedinoidei would be similar. These differences with respect to the type of prey targeted presumably relates to whether a ray species relies on its electric organs to subdue potential prey, or can forage effectively without recourse to producing electrical discharges.

Ambush predators tend to utilize intermittent feeding strategies, with individuals ingesting small numbers of relatively large prey [35]. The ability to stun large prey [13], [33] prior to ingestion involves either an electrical discharge [14], [38] as is the case for the Torpedinoidei, or the delivery of physical blows by the pectoral fins [39]–[42] as is the case for the Gymnuridae. By stunning their prey, ambush predators are able to target, handle and ingest large prey items which due to their size or mobility would not be accessible to other species [37], [39]. The ‘stun prior to ingestion’ feeding strategy is reflected in the cluster analysis which grouped the Gymnuridae and the majority of the Torpedinoidei together in the FISH trophic guild (Fig. 2). These species also had a high average *T*_L_ value of 4.01 (±0.08) which is comparable to that of most shark families (Table 3) [1].

Of the three filter-feeding *Mobula* species for which data were available, only the bentfin devil ray *Mobula thurstoni* (Lloyd, 1908) had a sufficiently large sample size for its inclusion in the cluster analysis and average *T*_L_ calculations. However, the diets of all three comprised over 99.5% EUPH (dominated by Euphausiids and Mysids) [43] indicating that the genus level *T*_L_ value of 3.25 is likely to be robust. Of note, a stable isotope-calculated *T*_L_ of 3.61 was reported for the lesser-devil ray *Mobula diabolus* (Shaw, 1804) [6] whose diet reportedly includes zooplankton as well as small pelagic fishes and crustaceans which would account for this species having a higher *T*_L_ value. It is noted though that *M. diabolus* is a non-valid synonym of the giant devil ray *Mobula mobular* (Bonnaterre, 1788) and that the presence of this species in the Indian Ocean is dubious. Given the small size of the specimens examined and the capture locality of the specimens examined [6], these may have been the pigmy devil ray *Mobula eregoodootenkee (Bleeker, 1859)*, the shortfin devil ray *M. kuhlii* (Müller & Henle, 1841) or the spinetail mobula *Mobula japanica* (Müller & Henle, 1841) [44]. Although data are currently lacking for these families, it seems likely that the majority of rays that utilise a filter feeding strategy would have a *T*_L_ value of a secondary consumer. This inference is supported by previous studies of filter feeding shark species which had *T*_L_ values of between 3.2 and 3.4 [1].

While feeding strategies were significant in determining what *T*_L_ a species, family or order occupied, other factors may have contributed to the results obtained. For instance, studies have shown that batoid diets can vary with body size [37], [45], maturity status [8], [46], geography [2], regional distributions [45] and seasonally [47]. Further, batoid species with overlapping ranges may partition food resources in order to reduce regional competition [48], [49]. This suggests that the *T*_L_ a species occupies at a regional level will vary through time and or growth [2]. To this extent, studies that assign a ‘fixed’ or ‘global’ *T*_L_ value to a species, family or order (current study, [1], [2]) do not take into account intraspecific *T*_L_ variability [2]. Given this, studies that focus on an individual or relatively few species are better suited for defining the role of elasmobranchs in regional food webs [1], [2]. This, however, remains an understudied aspect of batoid biology with relatively few analyses providing an overall *T*_L_ estimate for a species let alone information about the effects of, for example, season, locality or body size [3], [37], [46], [47], [49], [50].

While acknowledging the limitations of the current study, it is inherently difficult to account for diet variations across multiple species and multiple studies; even for smaller analyses. One of the primary reasons for this is a lack of consistency with respect to the criteria used to define prey importance and the definition of key parameters. For instance, individual indices such as frequency of occurrence [51] or volumetric contribution [39], [52] and compound indices such as the *I*_RI_ [16], [17], index of absolute importance [18], or index of preponderance [19] have all been used to define prey importance. As a consequence, inter- or intraspecific comparisons of dietary data across studies are often restricted to non-standardised dietary data or prey-importance rankings compiled using different criteria. Similarly, the stage of sexual maturity [37], [39], [53], sequential (equal and unequal) size bins [54], [55], disc length [53]–[55] and total length [56], [57] have all been used within the literature to define batoid size classes. This again makes it difficult to examine the influence of body size on batoid diets across multiple species or studies as the data may be lacking, often relates to different life-history stages and or to different size classes [2]. Evidently, one of the strengths of standardising the dietary data (Table S1) before calculating *T*_L_ estimates, is that it can easily compared across species, genera, families and orders irrespective of the methods used (Table 2, Table S1).

In the two previous studies [1], [2], *T*_L_ estimates were shown to have a positive, albeit moderate, correlation with the maximum total length of sharks [1] and skates [2]. In the current study, no significant correlation was detected between *T*_L_ and body size for either Myliobatoidei (disc length) or Torpedinoidei (total length). This result was largely attributed to the morphological variance displayed within each of the respective suborders [13], [14]. For example, disc width is approximately equal to the disc length in the Myliobatoidei families Dasyatidae and Urolophidae, whereas disc width in the Gymnuridae and Mobulidae is often more than double the disc length [12], [58]. Similarly, total length measurements vary considerably between genera of both suborders due to large variations in relative tail length [13]. As a consequence, disc width and total length are not necessarily good indictors of batoid body size. This problem is further compounded by the fact that disc length and arguably the best indicator of body size, body mass, is less reported in dietary analyses; especially for very large specimens such as *Manta*. Cortés [1] encountered a similar problem when examining the relationship between shark *T*_L_ and body size.

Outside of feeding strategies and body size, the *T*_L_ assigned to specific families or orders would more than likely have been influenced by morphological adaptations (Table 3). The two most obvious examples of this are the filter feeding family Mobulinae and the Torpedinoidei – the only elasmobranch suborder (or order) that possesses two well-developed electrical organs [14], [15]. There are however other less-prominent examples of morphological adaptations having a broader influence on batoid *T*_L_ estimates. For example, the mollusc trophic guild (Fig. 2) was dominated by the Myliobatinae and Rhinopterinae; species that possess flattened, well developed tooth plates on the upper and lower jaws [13], [33]. Formed through the fusing of teeth, tooth-plates aid the crushing of hard-shell prey-items such as oysters, whelks, clams and other bivalve molluscs [13], [33], [52]. As such, species within the Myliobatinae and Rhinopterinae are better able to process bivalves and other hard-shelled molluscs throughout life [59], [60]. In contrast, species from the families Dasyatidae, Urolophidae and Urotrygondidae, which have numerous rows of small overlapping teeth, may only be able to access or process this type of prey when they attain a large body size [35], [37], [59]. As a consequence, the presence of molluscs across a broader range of ray size classes may have increased the influence of this prey category when calculating the Myliobatinae and Rhinopterinae *T*_L_ estimates (Table 3). Conversely, the prominence of decapod and polycheate prey items in the diets of Dasyatidae, Urolophidae and Urotrygondidae species may reflect their preference for ‘softer’ prey items throughout much of their growth and development.

The primary purpose of the study was to examine the trophic relationships of Myliobatoidei and Torpedinoidei species. A multi-species analysis, this study provides a detailed synthesis of dietary data across a range of analytical techniques, sample sizes and sample areas. The results obtained help define trophic relationships of the Myliobatoidei and Torpedinoidei and highlight that these suborders comprise relatively diverse group of secondary and tertiary consumers whose diets are principally influenced by the feeding strategies employed; Continuous feeding or foraging; ambush predators and filter feeders. Secondary factors such as body size and morphological adaptations (e.g. possession of a crushing dentition) probably influence diet, and hence the *T*_L_ that an individual or species occupies, but the influence of these factors was beyond the scope of the current analysis. The results presented here significantly extend the comparative assessment of elasmobranch species’ feeding ecology, diet, and their trophic position within their environment. Further research is required though to determine how batoid trophic levels vary with development and the possible consequence of this with respect to the influence of individual species on regional food webs.

## Supporting Information

Table S1 **Standardized diets and trophic levels for species in the sub-orders Myliobatoidei and Torpedinoidei.**
(DOC)

Appendix S1 **Literature used to calculate the standardised diets and trophic levels (** ***T*** **_L_) for the suborders Myliobatoidei and Torpdinoidei.**
(DOC)
