# Supplementary material for: A Comparative Analysis of Feeding and Trophic Level Ecology in Stingrays (Rajiformes; Myliobatoidei) and Electric Rays (Rajiformes: Torpedinoidei)
Source: PLoS One. 2013 Aug 1;8(8):e71348. doi: 10.1371/journal.pone.0071348 (PMC3731297; doi:10.1371/journal.pone.0071348)
Supplement: Table S1 — Standardized diets and trophic levels for species in the sub-orders Myliobatoidei and Torpedinoidei. (DOC) [file pone.0071348.s001.doc]

**Table S1. S**tandardized diets and trophic levels for species in the sub-orders Myliobatoidei and Torpedinoidei.

|  | **Species** | ***N*** | ***n*** | **decA** | **ampH** | **eupH** | **cruS** | **molL** | **ceph** | **inv** | **fish** | **proT** | **polY** | **ELAS** | ***T*_L_** |
| --- | --- | --- | --- | --- | --- | --- | --- | --- | --- | --- | --- | --- | --- | --- | --- |
| **Myliobatoidei** |  |  |  |  |  |  |  |  |  |  |  |  |  |  |  |
| Dasyatidae | *Dasyatis akajei* | 1 | 156 | 30.82 | 0.00 | 2.83 | 22.96 | 1.41 | 1.01 | 0.00 | 31.38 | 0.00 | 9.60 | 0.00 | 3.72 |
|  | *Dasyatis americana* | 2 | 41 | 44.10 | 0.00 | 0.00 | 8.19 | 6.94 | 0.44 | 11.56 | 17.49 | 1.29 | 9.99 | 0.00 | 3.61 |
|  | *Dasyatis centroura* | 4 | 66 | 64.62 | 0.00 | 0.00 | 2.75 | 2.08 | 3.73 | 0.00 | 7.46 | 0.00 | 19.35 | 0.00 | 3.60 |
|  | *Dasyatis chrysonota* | 1 | 315 | 12.95 | 9.74 | 0.50 | 2.71 | 1.31 | 0.00 | 0.20 | 1.41 | 9.34 | 61.85 | 0.00 | 3.59 |
|  | *Dasyatis dipterura* | 2 | 527 | 49.09 | 0.50 | 0.00 | 1.59 | 29.42 | 0.00 | 0.20 | 1.27 | 0.00 | 17.43 | 0.50 | 3.43 |
|  | *Dasyatis guttata* | 1 | 239 | 81.78 | 0.08 | 5.32 | 2.30 | 8.77 | 0.00 | 0.61 | 0.53 | 0.00 | 0.59 | 0.00 | 3.47 |
|  | *Dasyatis lata* | 1 | 136 | 85.06 | 0.00 | 0.00 | 1.80 | 0.00 | 0.00 | 0.00 | 9.06 | 0.00 | 4.08 | 0.00 | 3.59 |
|  | *Dasyatis longa* | 2 | 235 | 53.53 | 0.00 | 0.00 | 19.03 | 0.66 | 0.10 | 0.07 | 26.45 | 0.00 | 0.16 | 0.00 | 3.69 |
|  | *Dasyatis margarita* | 1 | 11 | 48.90 | 9.07 | 0.00 | 3.04 | 23.32 | 0.00 | 0.00 | 4.80 | 0.00 | 10.88 | 0.00 | 3.52 |
|  | *Dasyatis marmorata* | 1 | 323 | 8.49 | 6.63 | 0.00 | 2.28 | 28.78 | 6.00 | 4.14 | 25.47 | 0.00 | 18.22 | 0.00 | 3.68 |
|  | *Dasyatis pastinaca* | 5 | 1265 | 67.35 | 1.29 | 0.00 | 5.38 | 6.04 | 3.99 | 0.34 | 14.09 | 0.00 | 1.32 | 0.19 | 3.63 |
|  | *Dasyatis say* | 1 | 34 | 68.19 | 0.00 | 0.00 | 0.01 | 0.00 | 4.36 | 0.00 | 7.75 | 0.00 | 19.69 | 0.00 | 3.62 |
|  | *Dasyatis tortonesei* | 1 | 666 | 22.59 | 0.00 | 0.00 | 5.99 | 15.82 | 14.83 | 1.87 | 32.22 | 0.00 | 5.01 | 1.67 | 3.80 |
|  | *Himantura alcockii* | 1 | 29 | 36.23 | 0.00 | 0.00 | 4.26 | 2.84 | 8.51 | 0.00 | 39.95 | 0.00 | 8.23 | 0.00 | 3.86 |
|  | *Himantura astra* | 2 | 192 | 64.42 | 0.16 | 0.00 | 25.65 | 0.46 | 0.21 | 2.32 | 5.39 | 0.05 | 1.34 | 0.00 | 3.53 |
|  | *Himantura bleekeri* | 1 | 67 | 38.38 | 0.00 | 0.00 | 28.86 | 13.73 | 0.00 | 0.00 | 18.38 | 0.00 | 0.65 | 0.00 | 3.56 |
|  | *Himantura fai* | 1 | 37 | 99.70 | 0.00 | 0.00 | 0.00 | 0.00 | 0.15 | 0.00 | 0.15 | 0.00 | 0.00 | 0.00 | 3.52 |
|  | *Himantura imbricata* | 2 | 328 | 42.54 | 11.70 | 12.79 | 4.88 | 0.25 | 0.02 | 0.00 | 3.07 | 0.00 | 24.74 | 0.00 | 3.60 |
|  | *Himantura uarnak* | 3 | 483 | 26.98 | 0.02 | 0.00 | 1.05 | 2.15 | 1.92 | 0.08 | 67.72 | 0.00 | 0.08 | 0.00 | 4.01 |
|  | *Himantura walga* | 1 | 159 | 95.53 | 0.00 | 0.00 | 0.41 | 0.00 | 0.00 | 0.00 | 4.06 | 0.00 | 0.00 | 0.00 | 3.55 |
|  | *Neotrygon annotata* | 1 | 77 | 24.80 | 0.20 | 0.00 | 0.10 | 0.20 | 0.00 | 0.20 | 1.49 | 1.79 | 71.22 | 0.00 | 3.58 |
|  | *Neotrygon kuhlii* | 2 | 195 | 41.19 | 0.11 | 0.00 | 12.22 | 0.56 | 0.00 | 0.11 | 9.12 | 0.00 | 36.69 | 0.00 | 3.60 |
|  | *Neotrygon picta* | 1 | 293 | 81.99 | 1.69 | 0.00 | 0.20 | 0.10 | 0.00 | 0.10 | 0.60 | 0.40 | 14.93 | 0.00 | 3.55 |
|  | *Pastinachus sephen* | 2 | 436 | 6.63 | 0.00 | 0.00 | 1.60 | 88.22 | 0.01 | 0.00 | 1.62 | 0.27 | 1.64 | 0.00 | 3.16 |
|  | *Pastinachus atrus* | 2 | 21 | 0.00 | 0.00 | 0.00 | 0.00 | 54.54 | 0.00 | 12.33 | 8.43 | 0.00 | 24.70 | 0.00 | 3.37 |
|  | *Pteroplatytrygon violacea* | 3 | 195 | 6.92 | 27.35 | 0.00 | 0.33 | 4.93 | 4.61 | 0.00 | 52.95 | 2.10 | 0.80 | 0.00 | 4.08 |
| Gymnuridae | *Gymnura altavela* | 2 | 151 | 0.00 | 0.00 | 0.00 | 12.77 | 7.41 | 4.85 | 0.00 | 74.97 | 0.00 | 0.00 | 0.00 | 4.05 |
|  | *Gymnura australis* | 1 | 62 | 0.03 | 0.01 | 0.00 | 0.00 | 0.05 | 0.00 | 0.00 | 99.79 | 0.01 | 0.11 | 0.00 | 4.24 |
|  | *Gymnura marmorata* | 1 | 433 | 1.63 | 0.00 | 0.00 | 0.00 | 2.77 | 0.93 | 0.00 | 94.67 | 0.00 | 0.00 | 0.00 | 4.20 |
|  | *Gymnura micrura* | 2 | 296 | 11.23 | 0.00 | 0.00 | 0.00 | 0.00 | 0.00 | 0.22 | 88.55 | 0.00 | 0.00 | 0.00 | 4.16 |
| Potamotrygonidae | *Paratrygon aiereba* | 1 | 21 | 8.42 | 0.00 | 0.00 | 0.00 | 0.00 | 0.00 | 8.32 | 83.27 | 0.00 | 0.00 | 0.00 | 4.12 |
|  | *Paratrygon sp* | 1 | 18 | 53.20 | 0.00 | 0.00 | 0.00 | 0.00 | 0.00 | 30.80 | 16.00 | 0.00 | 0.00 | 0.00 | 3.63 |
|  | *Plesiotrygon iwamae* | 1 | 4 | 1.00 | 0.00 | 0.00 | 0.00 | 0.00 | 0.00 | 0.00 | 0.00 | 0.00 | 0.00 | 0.00 | 3.52 |
|  | *Potamotrygon falkneri* | 1 | 10 | 0.28 | 0.00 | 0.00 | 0.00 | 99.45 | 0.00 | 0.00 | 0.28 | 0.00 | 0.00 | 0.00 | 3.10 |
|  | *Potamotrygon magdalenae* | 2 | 227 | 0.00 | 0.00 | 0.00 | 98.84 | 1.16 | 0.00 | 0.00 | 0.00 | 0.00 | 0.00 | 0.00 | 3.40 |
|  | *Potamotrygon motoro* | 3 | 165 | 64.51 | 0.00 | 0.00 | 0.03 | 1.88 | 0.00 | 11.26 | 22.29 | 0.00 | 0.03 | 0.00 | 3.67 |
|  | *Potamotrygon orbignyi* | 2 | 57 | 0.78 | 45.98 | 0.00 | 0.00 | 0.00 | 0.00 | 51.63 | 1.61 | 0.00 | 0.00 | 0.00 | 3.82 |
|  | *Potamotrygon scobina* | 1 | 8 | 0.18 | 0.82 | 0.00 | 0.00 | 0.00 | 0.00 | 0.00 | 0.00 | 0.00 | 0.00 | 0.00 | 4.06 |
| Urolophidae | *Urolophus cruciatus* | 2 | 117 | 21.60 | 42.56 | 0.00 | 12.31 | 0.18 | 0.77 | 0.92 | 0.00 | 0.00 | 21.66 | 0.00 | 3.81 |
|  | *Urolophus expansus* | 1 | 93 | 16.37 | 49.73 | 0.00 | 0.00 | 0.00 | 0.00 | 0.90 | 0.00 | 0.00 | 33.00 | 0.00 | 3.87 |
|  | *Urolophus halleri* | 3 | 484 | 30.25 | 17.18 | 0.78 | 18.07 | 12.31 | 0.02 | 2.80 | 1.46 | 0.35 | 16.78 | 0.00 | 3.58 |
|  | *Urolophus kapalensis* | 1 | 91 | 56.71 | 38.26 | 0.00 | 0.00 | 0.00 | 0.01 | 0.00 | 4.21 | 0.00 | 0.81 | 0.00 | 3.80 |
|  | *Urolophus lobatus* | 1 | 191 | 15.65 | 30.57 | 23.44 | 15.12 | 0.10 | 0.00 | 0.00 | 3.86 | 0.00 | 11.26 | 0.00 | 3.68 |
|  | *Trygonoptera mucosa* | 1 | 141 | 8.27 | 11.96 | 2.41 | 2.77 | 0.92 | 0.00 | 5.01 | 0.23 | 0.00 | 68.42 | 0.00 | 3.64 |
|  | *Urolophus paucimaculatus* | 1 | 196 | 24.46 | 32.18 | 14.95 | 14.17 | 0.65 | 0.52 | 0.10 | 0.23 | 0.00 | 12.74 | 0.00 | 3.69 |
|  | *Trygonoptera personata* | 1 | 150 | 13.95 | 22.24 | 14.18 | 14.94 | 0.68 | 0.00 | 2.92 | 0.00 | 0.00 | 31.10 | 0.00 | 3.63 |
|  | *Trygonoptera testacea* | 1 | 187 | 7.18 | 2.27 | 0.00 | 0.01 | 0.06 | 0.01 | 0.00 | 0.98 | 1.66 | 87.83 | 0.00 | 3.61 |
| Urotrygonidae | *Urotrygon aspidura* | 1 | 34 | 94.91 | 0.00 | 0.34 | 4.75 | 0.00 | 0.00 | 0.00 | 0.00 | 0.00 | 0.00 | 0.00 | 3.51 |
|  | *Urotrygon chilensis* | 1 | 192 | 15.90 | 9.37 | 0.00 | 0.01 | 0.00 | 0.00 | 0.00 | 0.81 | 0.00 | 73.92 | 0.00 | 3.65 |
|  | *Urotrygon microphthalmum* | 1 | 72 | 10.57 | 3.62 | 74.55 | 0.00 | 2.64 | 0.00 | 0.00 | 0.00 | 0.00 | 8.62 | 0.00 | 3.34 |
|  | *Urotrygon munda* | 3 | 517 | 63.16 | 0.93 | 0.00 | 26.83 | 0.00 | 0.01 | 0.00 | 9.07 | 0.00 | 0.00 | 0.00 | 3.56 |
|  | *Urotrygon nana* | 1 | 12 | 46.34 | 36.84 | 0.48 | 0.00 | 6.19 | 0.00 | 0.00 | 0.00 | 0.00 | 10.14 | 0.00 | 3.74 |
|  | *Urotrygon rogersi* | 4 | 513 | 63.13 | 4.80 | 0.09 | 14.67 | 0.12 | 0.00 | 0.36 | 0.48 | 0.00 | 16.35 | 0.00 | 3.55 |
|  | *Urotrygon venezuelae* | 1 | 56 | 91.83 | 0.00 | 0.00 | 0.00 | 0.00 | 0.00 | 8.17 | 0.00 | 0.00 | 0.00 | 0.00 | 3.52 |
| Myliobatinae | *Aetobatus flagellum* | 1 | 145 | 0.00 | 0.00 | 0.00 | 0.00 | 100.00 | 0.00 | 0.00 | 0.00 | 0.00 | 0.00 | 0.00 | 3.10 |
|  | *Aetobatis narinari* | 4 | 106 | 17.07 | 0.00 | 0.00 | 0.65 | 76.21 | 1.32 | 2.11 | 2.06 | 0.00 | 0.58 | 0.00 | 3.22 |
|  | *Aetomylaeus nichofii* | 1 | 52 | 0.00 | 0.00 | 0.00 | 30.08 | 18.80 | 0.00 | 0.00 | 43.61 | 0.00 | 7.52 | 0.00 | 3.72 |
|  | *Myliobatis aquila* | 3 | 1085 | 7.08 | 0.00 | 0.00 | 9.51 | 64.53 | 0.45 | 3.40 | 12.04 | 0.00 | 2.94 | 0.04 | 3.33 |
|  | *Myliobatis australis* | 1 | 173 | 19.74 | 9.41 | 0.00 | 8.04 | 40.62 | 1.37 | 2.51 | 2.99 | 0.00 | 15.32 | 0.00 | 3.44 |
|  | *Myliobatis californica* | 2 | 521 | 26.48 | 0.00 | 0.00 | 0.64 | 48.47 | 0.00 | 7.34 | 0.87 | 0.00 | 16.20 | 0.00 | 3.33 |
|  | *Myliobatis freminvillei* | 2 | 47 | 1.99 | 0.00 | 0.00 | 0.06 | 91.50 | 0.00 | 0.00 | 4.53 | 0.00 | 1.91 | 0.00 | 3.17 |
|  | *Pteromylaeus bovinus* | 2 | 568 | 8.43 | 0.00 | 0.00 | 1.12 | 41.62 | 16.08 | 2.75 | 26.31 | 0.00 | 3.10 | 0.60 | 3.65 |
| Mobulinae | *Mobula japonica* | 1 | 19 | 0.00 | 0.00 | 99.95 | 0.05 | 0.00 | 0.00 | 0.00 | 0.00 | 0.00 | 0.00 | 0.00 | 3.25 |
|  | *Mobula munkiana* | 1 | 3 | 0.00 | 0.00 | 99.66 | 0.34 | 0.00 | 0.00 | 0.00 | 0.00 | 0.00 | 0.00 | 0.00 | 3.25 |
|  | *Mobula thurstoni* | 1 | 52 | 0.00 | 0.01 | 99.68 | 0.31 | 0.00 | 0.00 | 0.00 | 0.00 | 0.00 | 0.00 | 0.00 | 3.25 |
| Rhinopterinae | *Rhinoptera bonasus* | 4 | 234 | 14.93 | 9.98 | 1.24 | 1.97 | 56.55 | 0.00 | 2.02 | 1.43 | 0.60 | 11.27 | 0.00 | 3.36 |
|  | *Rhinoptera steindachneri* | 2 | 42 | 0.59 | 0.03 | 0.48 | 0.01 | 16.30 | 0.00 | 78.92 | 3.02 | 0.00 | 0.65 | 0.00 | 3.51 |
| **Torpedinoidei** |  |  |  |  |  |  |  |  |  |  |  |  |  |  |  |
| Narcinidae | *Narcine brasiliensis* | 3 | 189 | 3.92 | 0.33 | 0.00 | 5.85 | 0.00 | 0.00 | 0.13 | 0.38 | 0.00 | 89.38 | 0.00 | 3.59 |
|  | *Narcine entemedor* | 2 | 369 | 4.38 | 0.02 | 0.00 | 1.02 | 15.25 | 0.00 | 0.25 | 13.90 | 0.00 | 65.17 | 0.00 | 3.61 |
|  | *Narcine tasmaniensis* | 1 | 72 | 8.36 | 34.40 | 0.00 | 0.04 | 0.00 | 0.00 | 0.01 | 0.00 | 0.00 | 57.19 | 0.00 | 3.79 |
| Torpedinidae | *Torpedo marmorata* | 4 | 323 | 0.03 | 0.11 | 0.00 | 0.00 | 0.00 | 0.62 | 0.00 | 98.24 | 0.00 | 0.01 | 1.00 | 4.24 |
|  | *Torpedo nobiliana* | 1 | 3 | 0.00 | 0.00 | 0.00 | 0.00 | 0.00 | 0.00 | 0.00 | 99.99 | 0.00 | 0.01 | 0.00 | 4.24 |
|  | *Torpedo torpedo* | 2 | 381 | 2.58 | 0.00 | 0.00 | 0.14 | 0.00 | 0.04 | 0.00 | 94.65 | 0.00 | 0.18 | 2.42 | 4.23 |
| Narkidae | *Heteronarce bentuviai* | 1 | 91 | 29.14 | 0.00 | 0.00 | 0.00 | 0.00 | 0.00 | 45.27 | 13.82 | 0.00 | 11.77 | 0.00 | 3.62 |
| Hypnidae | *Hypnos monopterygius* | 1 | 25 | 0.46 | 0.21 | 0.00 | 0.00 | 0.00 | 58.51 | 0.00 | 40.82 | 0.00 | 0.00 | 0.00 | 4.21 |

*N*, number of studies; *n*, number of stomachs sampled; *T*_L_, trophic level estimate. Refer to Table 1 for prey category definitions
