# Supplementary material for: A Comparative Analysis of Feeding and Trophic Level Ecology in Stingrays (Rajiformes; Myliobatoidei) and Electric Rays (Rajiformes: Torpedinoidei)
Source: PLoS One. 2013 Aug 1;8(8):e71348. doi: 10.1371/journal.pone.0071348 (PMC3731297; doi:10.1371/journal.pone.0071348)
Supplement: Appendix S1 — Literature used to calculate the standardised diets and trophic levels ( T L) for the suborders Myliobatoidei and Torpdinoidei. (DOC) [file pone.0071348.s002.doc]

**Appendix S1**. Literature used to calculate the standardised diets and trophic levels (*T*_L_) for the suborders Myliobatoidei and Torpdinoidei.

Abdel-Aziz SH (1994) Observations on the biology of the common Torpedo (*Torpedo torpedo*, Linnaeus, 1758) and marbled electric ray (*Torpedo marmorata*, Risso, 1810) from Egyptian Mediterranean Waters. Mar Freshw Res 45: 693–704.

Ajemin MJ, Powers SP (2012) Habitat-specific feeding by cownose rays (*Rhinoptera bonasus*) of the northern Gulf of Mexico. Environ Biol Fish 95(1): 79–97.

Almeida MP, Lins PMO, Charvet-Almeida P, Barthem RB (2010) Diet of the freshwater stingray *Potomotrygon motoro* (Chondrichthyes: Potamotrygonidae) on Marajó Island (Pará, Brazil). Braz J Biol 70(1): 155–162.

Babel JS (1967) Reproduction, life history, and ecology of the round stingray, *Urolophus halleri* Cooper. Fish Bull 137: 1–104. Available: <http://content.cdlib.org/view?docId=kt6t1nb1vn&brand=calisphere&doc.view=entire_text>. Accessed: 16 November 2012.

Bizzarro JJ (2005) *Fishery Biology and Feeding Ecology of Rays in Bahia Almejes, Mexico*. MS Thesis, California State University, San Francisco, CA, USA.

Bornatowski H, Abilhoa V, Freitas MO (2006) Sobre a alimentação de *Narcine brasiliensis* na baía de ubatuba-enseada, São Francisco do sul, Santa Catarina, Brasil. Estud Biol Univ Cat Parana 28(62): 57–60.

Bowman RE, Stillwell CE, Michaels WL, Grosslein MD (2000) Food of Northwest Atlantic fishes and two common species of squid. Woods Hole, Massachusetts: U S Dept. of Commerce, National Oceanic and Atmospheric Administration. 156 p.

Braganca AJM (2004) Preliminary observation on the feeding of the freshwater stingrays *Potamotrygon orbignyi, Potamotrygon scobina* and *Plesiotrygon iwamae* (Chondrichthyes: Potamotrygonidae) in the Cotijuba Island Region - Pará, Brazil. In Martin RA, MacKinlay D, editors. Biology and Conservation of Freshwater Elasmobranchs Symposium Proceedings. Brazil: International Congress on the Biology of Fish. pp. 41-51.

Capapé C (1975) Contribution a la biologie des Dasyatidae des côtes Tunisiennes II – *Dasyatis pastinaca* (Linne, 1758): regime alimentaire. Ann Inst Michel Pacha 8: 1–15.

Capapé C (1976) Etude du régime alimentaire de la Mourine vachette, *Pteromylaeus bovinus* (Geoffroy Saint-Hilaire, 1817) (Pisces, Myliobatidae) des côtes Tunisiennes. Cons Int Explor Mer 37(3): 214–220.

Capapé C (1976) Étude du régime alimentaire de l’Aigle de mer, *Myliobatis Aquila* (L., 1758) des côtes Tunisiennes. Cons Int Explor Mer 37(1): 29–35.

Capapé C (1978) Contribution a la biologie des Dasyatidae des cotes Tunisiennes IV – *Dasyatis tortonesei* Capapé 1975: regime alimentaire. Arch Inst Pasteur Tunis 55(3): 359–369.

Capapé C (1985) Donées générales sur le régime alimentaire des Dasyatidae (Pisces, Selachii). Arch Inst Pasteur Tunis 62(3): 299–304

Capapé C (1986) Donées générales sur le régime alimentaire des Gymnuridae et des Mobulidae (Pisces, Selachii). Arch Inst Pasteur Tunis 63(2–3): 241–246.

Capapé C, Zaouali J (1992) Le regime alimentaure de la pastenagye marbrée, *Dasyatis marmorata* (Pisces, Dasyatidae), des eauz Tunisiennes. Vie Milieu 42: 269–276.

Capapé C, Crouze S, Clémet C, Vergne Y, Guélorget O (2007) Diet of the marbled electric ray *Torpedo marmorata* (Chondrichthyes: Torpedinidae) off the Languedocian Coast (Southern France, Northern Mediterranean). Ann Ser Hist Nat 17(1): 17–22.

Collins AB, Heupel MR, Hueter RE, Motta PJ (2007) Hard prey specialists or opportunistic generalists? An examination of the diet of the cownose ray, *Rhinoptera bonasus*. Mar Freshw Res 58: 135–144.

Dale JJ, Wallsgrove NJ, Popp BN, Holland KN (2011) Nursery habitat use and foraging ecology of the brown stingray *Dasyatis lata* determined from stomach contents, bulk and amino acid stable isotopes. Mar Ecol Prog Ser 433: 221–236.

Darracott A (1977) Availability, morphometrics, feeding and breeding activity in a multispecies, demersal fish stock of the Western Indian Ocean. J Fish Biol 10: 1–16.

Devadoss P (1978) On the food of rays, *Dasyatis uarnak* (Forskål), *D. alcockii* (Annandale) and *D. sephen* (Forskål). Indian J Fish 25: 9–13.

Devadoss P (1983-84) Further observations on the biology of the sting ray, *Dasyatis imbricatus* (Schneider) at PortoNovo. Matsya 9-10: 129–134.

Ebert DA, Cowley PD (2003) Diet, feeding behaviour and habitat utilisation of the blue stingray *Dasyatis chrysonata* (Smith, 1828) in South African waters. Mar Freshw Res 54: 957–965.

Ferreira LC, Vooren CM (2012) Diet of the lesser electric ray *Narcine brasiliensis* (Olfers, 1831) (Elasmobranchii, Narcinidae) in southern Brazil. PANAMJAS. 7(1): 37–44.

Flores-Ortega JR, Godínez-Dominguez E, González-Sansón G, Rojo-Vázquez, JA, Corgos A, Morales-Jáuregui A (2011) Feeding habits of three round stingrays Rajiformes: Urotrygonidae) in the central Mexican Pacific. Cienc Mar 37(3): 279–292.

Gianeti MD (2011) *Reprodução, alimentação, idade e crescimento de Dasyatis guttata (Bloch & Schneider, 1801) (Elasmboranchii; Dasyatidae) na região de Caiçcara do Norte – RN*. Doutor em Ciências, Universidade de Sao Paulo.

Gilliam D, Sullivan KM (1993) Diet and feeding habits of the southern stingray *Dasyatis americana* in the central Bahamas. Bull Mar Sci 52: 1007–1013.

Goitein R, Torres FS, Signorini CE (1998) Morphological aspects related to feeding of two marine skates *Narcine brasiliensis* Olfers and *Rhinobatos horkelli* Müller & Henle. Acta Sci., Biol 20(2): 165–169.

Goldman AM (2007) Aspects of the biology of the coffin ray, *Hypnos monopterygius* (Shaw & Nodder, 1795), (Chondrichthyes; Hypnidae). Honours thesis, University of Queensland, Brisbane, Australia. 65 p.

Gray AE, Mulligan TJ, Hannah RJ (1997) Food habits, occurrence, and population structure of the bat ray, *Myliobatis californica*, in Humboldt Bay, California. Environ Biol Fish 49: 227–238.

Grijalba-Bendeck M, Polo-Silva C, Acevedo K, Moreno F, Mojica D (2012) Aspectos tróficos y reproductivos de algunos batoideos capturados en Santa Marta, Mar Caribe de Colombia. LAJAR 40(2): 300–315.

Guzmán AB (2011) *Demografía de la raya chilena (Urotrygon chilensis) (Günther, 1872) presente en el Golfo de Tehuantepec México*. Maestria en Ciencias. Instituto Politécnico Nacional. 82 p.

Hess PW (1961) Food habits of two dasyatid rays in Delaware Bay. Copeia 1961**:** 239–241.

Ismen A (2003) Age, growth, reproduction and food of common stingray (*Dasyatis pastinaca* L., 1758) in Iskenderun Bay, the eastern Mediterranean. Fish Res 60: 169–176.

Jacobsen IP, Johnson JW, Bennett MB (2009) Diet and reproduction in the Australian butterfly ray *Gymnura australis* from northern and north-eastern Australia. J Fish Biol 75: 2475–2489.

Jacobsen IP, Bennett MB (2011) Life history of the blackspotted whipray *Himantura astra*. J Fish Biol 78: 1249–1268.

Jacobsen IP, Bennett MB (2012) Feeding ecology and dietary comparisons among three sympatric *Neotrygon* (Myliobatoidei: Dasyatidae) species. J Fish Biol 80: 1580–1594.

Jardis I, Šantić M, Pallaoro A (2004) Diet composition of the eagle ray, *Myliobatis aquila* (Chondrichthyes: Myliobatidae), in the Eastern Adriatic Sea. Cybium 28(4): 372–374.

Karachle PK, Stergiou KI (2010) Food and feeding habits of nine elasmobranch species in the N Aegean Sea. CIESM 39: 553.

Lipej L, Mavrič B, Paliska D, Capapé C (2012) Feeding habits of the pelagic stingray *Pteroplatytrygon violacea* (Chondrichthyes: Dasyatidae) in the Adriatic Sea. J Mar Biol Assoc.

Lobato C, da Silva Z (2003) Habito alimentar de *Urotrygon microphthalmum* Delsman, 1941 (Elasmobranchii, Urolophidae) En Tutoia Maranhao. Bol Lab Hidrobiol 16: 47–54.

Londoño ANJ (2009) *Tesis Doctoral Estudio de la biologia trofica de cinco especies de peces bentonicos de la costa de cullera. Relaciones con la acumulacion de metales pesados*. Tesis, Universidad politecnica de Valencia, Valencia.478 p.

López-García J, Navia AF, Mejía-Falla PA, Rubio EA (2012) Feeding habits and trophic ecology of *Dasyatis longa* (Elasmobranchii: Myliobatiformes): sexual, temporal and ontogenetic effects. J Fish Biol 80: 1563–1579.

Marshall AD, Kyne PM, Bennett MB (2008) Comparing the diet of two sympatric urolophid elasmobranchs (*Trygonoptera testacea* Müller & Henle and *Urolophus kapalensis* Yearsley & Last): evidence of ontogenetic shifts and possible resource partitioning. J Fish Biol 72: 883–898.

Maurer RO, Bowman RE (1975) *Food Habits of Marine Fishes of the Northwest Atlantic – data report*. Woods Hole, Massachusetts: Northeast Fisheries Center National Marine Fisheries Service National Oceanic and Atmospheric Administration. 102 p.

Mavrič B, Jenko R, Makovec T, Lipej L (2004) On the occurrence of the pelagic stingray, *Dasyatis violacea* (Bonaparte, 1832), in the Gulf of Trieste (Northern Adriatic). Ann Ser Hist Nat 14: 181–186.

Navarro-Ganzáles JA, Bóhorquez-Herrera J, Navia AF, Cruz-Escalona VH (2012) Diet composition of batoids on the continental shelf off Nayarit and Sinaloa, Mexico. Cienc Mar 38(2): 347–362.

Navia AF, Mejía-Falla PA, Giraldo A (2007) Feeding ecology of elasmobranch fishes in coastal waters of the Colombian Eastern Tropical Pacific. BMC Ecology 7: 1–10.

Navia AF, Torres A, Mejía-Falla PA, Giraldo A (2011) Sexual, ontogenetic, temporal and spatial effects on the diet of *Urotrygon rogersi* (Elasmobranchii: Myliobatiformes). J Fish Biol 78(4): 1213–1224.

Notarbatolo-di-Sciara G (1988) Natural history of the rays of the genus *Mobula* in the Gulf of California. Fish Bull 66(1): 45–66.

Omotosho JS (1996) Comparative analysis of the chemical composition of *Dasyatis margarita* (Gunther) with *Tilapia zillii* (Gervias) and *Clarias gariepinus* (Buchell) from Nigerian waters. Biosci Res Comm 8(3): 215–219.

Omotosho JS, Oyebanji MO (1997) *On Some Aspects of the Biology of Dasyatis margarita (Gunther) from Nigeria Continental Shore.* University of Ilorin, Nigeria. Available:

[http://www.unilorin.edu.ng/publications/omotosho/On%20some%20aspects%20of%20the%20Biology%20of%20Dasyatis%20Margarita.pdf](http://www.unilorin.edu.ng/publications/omotosho/On some aspects of the Biology of Dasyatis Margarita.pdf). Accessed 7 November 2012.

Oribhabor BJ, Ogbeibu AE (2012) The food and feeding habits of fish species assemblage in a Niger Delta mangrove creek, Nigeria. J Fish Aquat Sci 7(2): 134–149.

Patokina FA, Litvinov FF (2005) Food composition and distribution of elasmobranchs on the shelf and upper slope of the eastern central Atlantic. ICES CM 2005 / N:26 22 p.

Platell ME, Potter IC, Clarke KR (1998) Resource partitioning by four species of elasmobranches (Batoidea: Urolophidae) in coastal waters of temperate Australia. Mar Biol 131: 719–734.

Raje SG (2003) Some aspects of biology of four species of rays off Mumbai water. Indian J Fish 50: 86–96.

Raje SG (2007) Some aspects on the biology of *Himantura bleekeri* (Blyth) and *Amphotistius imbriactus* (Schneider) from Mumbai. Indian J Fish 54(2): 235–238. Bottom of Form

Top of Form

Bottom of Form

Top of Form

Bottom of Form

Ramos-Socha HB, Grijalba-Bendeck M (2011) Bioecolgía de la rays de agua dulce *Potamotrygon magdalenae* (Duméril, 1865) (Myliobatiformes) en la Ciénaga De Sabayo, Guaimaral, Columbia. Rev U D C A Act & Div Cient 14(2): 109–118.

Randall, J. H. (1967). Food habits of reef fishes of the West Indies. Stud Trop Oceanogr 5: 665–847.

Romanelli M, Consalvo I, Vacchi M, Finoia G (2006) Diet of *Torpedo torpedo* and *Torpedo marmorata* in a coastal area of Central Western Italy (Mediterranean Sea). Mar Life 16: 21–30.

Saglam H, Orhan AK, Kutlu S, Aydin I (2010) Diet and feeding strategy of the common stingray *Dasyatis pastinaca* (Linnaeus, 1758) on the Turkish coast of southeastern Black Sea. Cah Biol Mar 51: 37–44.

Salini JP, Blaber SJM, Brewer DT (1994) Diets of trawled predatory fish of the Gulf of Carpentaria, Australia with particular reference to predation on prawns. Mar Freshw Res 45: 397–411.

Šantić M, Paladin A, Agović A (2011) Diet of the common stingray, *Dasyatis pastinaca* (Chondrichthyes: Dasyatidae) in the eastern Adriatic Sea. Cah Biol Mar 52: 319–356.

Schluessel V, Bennett MB, Collin SP (2010) Diet and reproduction in the white-spotted eagle ray *Aetobatus narinari* from Queensland, Australia and the Penghu Islands, Taiwan. Mar Freshw Res 61: 1278–1289.

Shibuya A, Araújo MLG, Zuanon JAS (2009) Analysis of stomach contents of freshwater stingrays (Elasmobranchii, Potamotrygonidae) from the middle Negro River, Amazonas, Brazil. PANAMJAS 4(4): 466–475.

Silva TB, Uieda VS (2007) Preliminary data on the feeding habits of the freshwater stingrays *Potomotrygon falkneri* and *Potamotrygon motoro* (Potamotrygonidae) from the Upper Paraná River basin, Brazil. Biota Neotropica 7(1): 221–226.

Smith JW, Merriner JV (1985) Food habits and feeding behaviour of the cownose ray, *Rhinoptera bonasus,* in Lower Chesapeake Bay. *Estuaries* 8(3): 305–310.

Sommerville E, Platell ME, White WT, Jones AA, Potter IC (2010) Partitioning of food resources by four abundant co-occurring elasmobranch species: relationships between diet and both body size and season. Mar Freshw Res 62: 54–65.

Struhsaker P (1969) Observations on the biology and distribution of the thorny stingray, *Dasyatis centroura* (Pisces: Dasyatidae). Bull Mar Sci 19: 456–458.

Talent LG (1982) Food habits of the gray smoothhound, *Mustelus californicus*, the brown smoothhound *Mustelus henlei,* the shovelnose guitarfish, *Rhinobatos productus*, and the bat ray *Myliobatis californica*, in Elkhorn Slough, California. Calif Fish Game 68(4): 224–234.

Taniuchi T, Shimizu M (1993) Dental sexual dimorphism and food habits in the stingray *Dasyatis akajei* from Tokyo Bay, Japan. Nippon Suisan Gakk 59(1): 53–60.

Treloar MA, Laurenson LJB (2006) Preliminary observations on the reproduction, growth and diet of *Urolophus cruciatus* (Lacépède) and *Urolophus expansus*, McCulloch (Urolophidae) in Southeastern Australia. Proc R Soc Vic 117: 341–347.

Valadez-González C (2000) Hábitos alimentarios de las rayas (Chondrichthyes: Rajiformes) capturadas en la plataforma continental de Jalisco y Colima, México Septiembre 1997- Agosto de 1998. Tesis de Maestría en Biología, Universidad de Colima, Colima, Mexico.

Valadez-González C, Aguilar B, Hernández S (2001) Hábitos alimentarios de la raya *Urobatis halleri* (Cooper 1863) (Chondrichthyes: Urolophidae) capturada en la plataforma continental de Jalisco y Colima, México. Cienc Mar 27: 91–104.

Valls M, Quetglas A, Ordines F, Moranta J (2007) Feeding ecology of demersal elasmobranchs from the shelf and slope off the Balearic Sea (western Mediterranean). Sci Mar 75(4): 633–639.

Vaudo JJ, Heithaus MR (2011) Dietary niche overlap in a nearshore elasmobranch mesopredator community. Mar Ecol Prog Ser 425: 247–260.

Véras DP, Vske T, Hazin FHV, Lessa RP, Travassos E, Tolotti MT, Barbosa TM (2009) Stomach contents of the pelagic stingray (*Pteroplatytrygon violacea*) (Elasmbranchii: Dasyatidae) from the tropical Atlantic. Braz J Oceanogr 57(4): 339–343.

Woodland RJ, Secor DH, Wedge ME (2011) Trophic resource overlap between small elasmobranchs and sympatric teleosts in Mid-Atlantic Bight nearshore habitats. Estuar Coast 34: 391–404.

Yamaguchi A, Kawahara I, Shirou I (2005) Occurrence, growth and food of longheaded eagle ray, *Aetobatus flagellum*, in Ariake Sound, Kyushu, Japan. Environ Biol Fish 74: 229–238.

Yeldan H, Avsar D, Manasirh M (2009) Age, growth and feeding of the common stingray (*Dasyatis pastinaca,* L., 1758) in the Cilician coastal basin, northeastern Mediterranean Sea. J Appl Ichtyol 25 (Suppl. 1): 98–102.

Yick JL, Tracey SR, White RWG (2011) Niche overlap and trophic resource partitioning of two sympatric batoids co-inhabiting an estuarine system in southeast Australia. J Appl Ichtyol 27: 1272–1277.

Yokota L (2010) *Taxonomia, Biologia Reproductiva e Dieta Da Raia Borboleta, Gymnura micrura (Myliobatiformes: Elasmobranchii)*. Pós-Graduação em Ciências Biolôgicas, Universidade Estadual Paulista (UNESP).
